# A Model for World-Class 10,000 m Running Performances: Strategy and Optimization

**DOI:** 10.3389/fspor.2020.636428

**Published:** 2021-01-20

**Authors:** Quentin Mercier, Amandine Aftalion, Brian Hanley

**Affiliations:** ^1^Centre d'Analyse et de Mathématique Sociales, CNRS UMR-8557, Ecole des Hautes Etudes en Sciences Sociales, Paris, France; ^2^Carnegie School of Sport, Leeds Beckett University, Leeds, United Kingdom

**Keywords:** athletics, coaching, pacing, race tactics, track and field

## Abstract

The distribution of energetic resources in world-class distance running is a key aspect of performance, with athletes relying on aerobic and anaerobic metabolism to greater extents during different parts of the race. The purpose of this study is to model 10,000 m championship performances to enable a deeper understanding of the factors affecting running speed and, given that more than half the race is run on curves, to establish the effect of the bends on performance. Because a limitation of time split data is that they are typically averaged over 100-m or 1,000-m segments, we simulate two 10,000 m runners' performances and thus get access to their instantaneous speed, propulsive force and anaerobic energy. The numerical simulations provide information on the factors that affect performance, and we precisely see the effect of parameters that influence race strategy, fatigue, and the ability to speed up and deal with bends. In particular, a lower anaerobic capacity leads to an inability to accelerate at the end of the race, and which can accrue because of a reliance on anaerobic energy to maintain pace in an athlete of inferior running economy. We also see that a runner with a worse running economy is less able to speed up on the straights and that, in general, the bends are run slower than the straights, most likely because bend running at the same pace would increase energy expenditure. Notwithstanding a recommendation for adopting the accepted practices of improving aerobic and anaerobic metabolism through appropriate training methods, coaches are advised to note that athletes who avoid mid-race surges can improve their endspurt, which are the differentiating element in closely contested championship races.

## Introduction

The 10,000 m race is the longest track event held as part of the Olympic Games and World Athletics Championships. As an endurance event, the distribution of energetic resources is of prime importance in achieving one's best finishing time, which is theoretically most likely to be achieved with an even pacing profile as seen in cycling time trials (Padilla et al., 2000). Indeed, the world record for the men's 10,000 m running event was recently set not only with the aid of specially-prepared pacemakers but also with a lighting system, the Wavelight pacing technology, which was programmed to show the even pace of the previous record (World Athletics, 2020a). However, championship racing, where the primary aim of the world's best athletes is to win, regardless of finishing time, features much more variable pacing that reflects tactical decision-making (Casado et al., 2020a). Even when world-class athletes do not run at their maximum sustainable speed in racing, the stresses on their physiological systems still come at a considerable energetic cost. Some of the most important factors that affect performance in distance running are oxygen uptake (*VO*_2_), particularly relative to an individual's maximal oxygen update (Jones et al., 2020), running economy (Lucia et al., 2008) and anaerobic reserve, which is reflected most clearly in the faster speeds experienced during the endspurt (Billat et al., 2003). Most of a 10,000 m race is run at a pace below the critical speed, which is the speed above which finite, predominantly non-oxidative exercise is performed (Burnley and Jones, 2010). In a sense, the objective of a successful pacing strategy is to deplete all possible energy stores (whether by aerobic or anaerobic metabolism) by the end of the race, but not too early that catastrophic deceleration occurs (Foster et al., 2004; Thiel et al., 2012). Hettinga et al. (2019) used 100-m split data to show that Olympic and World Championship 10,000 m male athletes continually changed pace throughout the race, with the best athletes able to achieve higher speeds than the rest of the field from 8,000 m onward. However, no physiological measures were possible in their analysis and contributing factors such as *VO*_2_ are instead estimated for field-based exercise using mathematical analyses (Péronnet and Thibault, 1989). A novel study that models the effects of the factors that affect performance in the 10,000 m will therefore improve our understanding of what differentiates better performances and inform coaches of appropriate training practices.

The 10,000 m track race comprises 50 straight sections and 50 bends, although the length of the bend and the straight are not equal; in fact, on a standard track, the bends are 116 m long and the straights are 84 m long (World Athletics, 2019a). That the straight part of the track is shorter can be seen from the way in which the 100 m sprint race has its start line on an extension from the rest of the track. The effect of running on bends has been analyzed for short distance races (Quinn, 2009; Ohnuma et al., 2018; Aftalion and Martinon, 2019; Churchill et al., 2019; Aftalion and Trélat, 2020), but never for the 10,000 m, despite the potential impact of 50 bends on running performance. Many 10,000 m competitors in major championships are unused to running such long-distance races over multiple laps, as they instead mostly compete in shorter track races, on roads, or in cross country events. Indeed, in the 2017 World Championships men's 10,000 final, one of the competitors had never run that distance on the track before, having qualified for the championships as one of the top 15 finishers in that year's World Cross Country Championships (Hanley et al., 2018). Notwithstanding that athletes might run the bends effectively slower than the straights because of taking a racing line away from the inside kerb, the centrifugal forces and reduced horizontal propulsion experienced during bend running might also affect the speeds attained, as in sprinting (Judson et al., 2019). To maintain constant metabolic energy expenditure, athletes must run slower on the bends; by the same token, if they wish to maintain an even pace, they must increase energy expenditure (Taboga and Kram, 2019). A tactical decision to run wide on the bends in an Olympic 5,000 m final was shown to affect the medal positions (Jones and Whipp, 2002), and so our study that includes analysis of the effects of bends in the longer 10,000 m race will be useful to athletes and coaches in establishing whether bend running is a skill that requires development in training for that event.

To date, studies on pacing in the 10,000 m event have relied on 1,000-m split times (Filipas et al., 2018) or 100-m split times (Thiel et al., 2012; Hettinga et al., 2019), and have shown that changes in pace occur regularly during championship racing, especially during the final 1,000 m. Indeed, achieving a high finishing position in 10,000 m racing is associated with the ability to produce or withstand high pace variability, and more so with the capability of producing a fast final endspurt (Renfree et al., 2020). However, even the higher-resolution data used in previous research are restricted to mean speeds over 100-m distances that could hide some interesting pacing phenomena and prevent a fuller understanding of instantaneous speed and its changes. In the present study, instead of using statistical analyses of 100-m mean speeds, we choose to analyze a select sample of athletes individually and fit a mathematical model that closely resembles their pacing profiles. This process gives access to the instantaneous speed and thus helps us explain the physiological parameters that influence race strategy, fatigue, or the ability to speed up, particularly at the end of the race. Using a deterministic model means that for each race and each athlete, new specific computations are involved, and so in this study we focus on a single race. Furthermore, because the process adopted is a deterministic model, rather than a statistical model or experiment, we do not present a hypothesis. The aim of this study is to model 10,000 m world-class racing performances to allow for a deeper understanding of the factors that affect running speed and to establish the effect of the bends on performance.

## Materials and Methods

Split time data for each 100-m segment of the men's 10,000 m final at the 2017 IAAF World Championships were obtained from Hanley et al. (2018). Athlete finishing times and personal best times (PBs) (min:s) were also obtained from that report. The race start-time temperature was 20°C and the humidity was 64% (Hanley et al., 2018). To calculate mean speed (m/s) from the running split data, we divide each 100-m distance by time taken. We fit a mathematical model onto the 100-m mean speed data from the race winner (finishing time: 26:49.51) so that realistic constraints are applied that allow insights into the relative effects of different physiological characteristics. We also use the 100-m mean speed data from the 6th-place finisher for our models (finishing time: 26:57.77), based on similarities in these two athletes' racing conditions. For example, the 6th-place runner started on a similar part of the stagger (22nd athlete from the inside) to the winner (20th athlete from the inside), and so there was little or no difference in the effect of the first bend between these two athletes.

**Figure 1:**
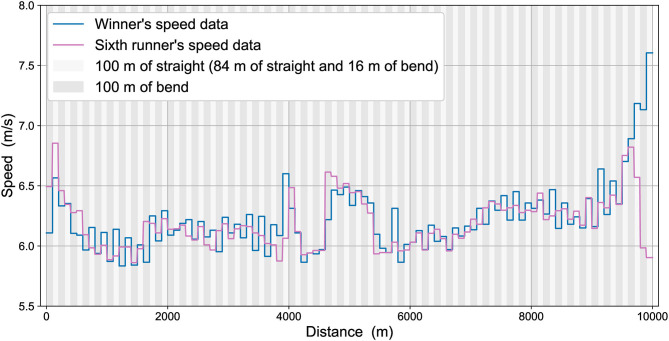
The race data for the winner's speed (blue line) and 6th-place runner's speed (pink line).

The 100-m split mean data for the winner's speed and 6th-place runner's speed are shown in Figure 1. The two athletes were never more than 3.23 s apart (~19 m apart, based on the mean running speed for each 100-m segment), except during the last lap where the winner was 4.47 s ahead (~29 m apart) with 100 m remaining, and finished the race 8.26 s ahead (~56 m apart). The mean absolute difference between them during the first 9,600 m was 1.39 s (±0.97 s), equivalent to about 9 m apart. The winner's PB before the competition was 26:46.57, slightly quicker than the 6th-place athlete's PB of 26:52.65. These athletes had the quickest and fourth-quickest PBs of all starters before the race. Both athletes undertook a strong acceleration at the very beginning of the race and at its end, with the middle part not at a constant speed, but included some tactical elements, in that running pace was elevated between ~4,000 and 6,000 m (Figure 1). Whereas, the race winner generally increased pace throughout the last 1000 m, and whose last 100-m segment was his quickest of all, the 6th-place athlete could follow this increased pace until about 9,600 m only, after which his pace decreased so considerably that his last 100-m was one of his slowest. With considerations of these different tactical approaches at different stages of the race, our aim is to understand the effect of the physiology, mechanics and optimization of effort on these different sections of the race.

### Description of the Track

The 2017 World Championship 10,000 m final was run on a standard athletics track 400 m long, made up of straights of 84 m and half circles of radius 36.5 m, acknowledging that the length of the track (in the inside lane) is measured 0.30 m from the kerb, so that the curvature of the track is either zero (in the straights) or 1/36.5 (in the bends) (World Athletics, 2019a). 10,000 m races begin on the first bend using a staggered start, and then the 116-m length of the bends and the 84-m length of the straights alternate. Because the split time data used were recorded every 100 m, the data corresponding to the straights therefore actually include 16 m of bend running.

### Deterministic Model

The aim of this study is to model 10,000 m world-class racing performances to allow for a deeper understanding of the factors that affect running speed. Rather than using statistical analyses of mean data over 100-m or 1,000-m segments, we want to fit a model to replicate actual athletes' data so that instantaneous speeds can be calculated.

The model (Aftalion and Bonnans, 2014; Aftalion, 2017; Aftalion and Martinon, 2019; Aftalion and Trélat, 2020) yields an optimal control problem based on a system of coupled ordinary differential equations for the instantaneous speed *v(s)*, the propulsive force per unit of mass *f(s)*, and the anaerobic energy *e(s)*, where *s* is the distance from start. The system relies on Newton's second law of motion and the energy balance that takes into account the aerobic contribution *VO*_2_, the anaerobic contribution *e(s)* and the power developed by the propulsive force. Simulations require numerical values for the athletes' parameters. Based on previous research on the world's best athletes and the proportion of maximal oxygen uptake that occurs in elite-standard 10,000 m running, we assume *VO*_2*max*_ is 85 mL/kg/min and, in a championship race (rather than a more evenly paced world record), that the athletes run at roughly 85% of this value (Péronnet and Thibault, 1989; Joyner, 1991; Saltin et al., 1995; Billat et al., 2003; Lucia et al., 2008). The other key physiological parameters that influence pacing are:

- the maximal propulsive force per unit of mass *f*_*M*_,
- the global friction coefficient τ that encompasses all kinds of friction, both from joint and track. In total, *f*_*M*_ τ is the maximal speed,
- the maximal decrease rate and increase rate of the propulsive force, which is related to motor control: an athlete cannot stop or start his effort instantaneously, but needs some time or distance to do so. This is what our control parameters *u*_−_ and *u*_+_ provide,
- the total anaerobic energy or maximal accumulated oxygen deficit *e*^0^,
- the *VO*_2_ profile as a function of distance. This is a curve σ*(s)* where *s* is the relative distance from the start but in fact, in the model, it is a curve σ*(e(s))* where *e(s)* is the remaining anaerobic energy. We refer the reader to Aftalion (2017), Aftalion and Martinon (2019), Aftalion and Trélat (2020) for more details on the model.

These parameters *e*^0^*, u*_−_*, u*_+_*, f*_*M*_, τ and the function σ are not measured for each athlete but are estimated through a computation to fit the data. More precisely, they are identified for a specific race and athlete (i.e., the winner and 6th-place finisher in the 10,000 m final in 2017). For fixed values of the parameters, the optimal control problem is solved using Bocop, an open license software developed by Inria-Saclay France (Bonnans et al., 2014). In total, we minimize the error between a single optimization simulation and data for a wide range of parameters to identify those that best match the data.

A crucial piece of information to be included in our model is the centrifugal force on the bends: it does not act as such in the equation of motion but limits the propulsive force *f(s)* through a constraint that yields a decrease in the effective propulsive force on the bends:

$$\begin{array}{l} {f\left(s\right)}^{2}+\frac{{v\left(s\right)}^{4}}{{R\left(s\right)}^{2}}\leq f_{M}^{2} \end{array}$$

where *R(s)* is the radius of curvature at distance *s* from the start and *f*_*M*_ is the maximal force that the runner can exert.

Because competition officials do not record wind speeds for races longer than 200 m (World Athletics, 2019b), no precise wind data were available for the 10,000 m final. Given the wind reading was +0.3 m/s in the preceding race (World Athletics, 2020b) (the last heat in the first round of the men's 100 m), we considered the effect of wind to be negligible in terms of its effects on the runners' speed or strategy for the 10,000 m final. Concerning our model, it is not an issue to include any wind effects (Pritchard, 1993; Quinn, 2003), even allowing for the bends, provided there are precise data on wind speed and direction for the duration of the race (which is not presently the case for championship races). The wind adds an extra friction term in the law of motion; this term depends on air density (and therefore altitude), on the frontal area of the athlete in the direction of the wind (which changes during the race with wind direction) and the drag coefficient. Thus, the wind effect can be included in the equations, but for a 10,000 m event it is not going to affect performance because neither running speed nor wind speed are fast enough.

## Results

### The Winner's Race

From the winner's speed data every 100 m, we identify the physiological parameters of this athlete and therefore we are able to compute his optimal speed. We show the instantaneous speed because it allows us to see the variations better. To compute the mean speed, we want to match the split data, therefore we compute the time *T*_*k*_ for the *k*^*th*^ segment of 100 m, which is:

$$\begin{array}{l} T_{k}=\int_{100\left(k-1\right)}^{100k}\frac{1}{v\left(x\right)}dx. \end{array}$$

Thus, the mean speed for the *k*^*th*^ segment is *v*_*k*_ = *100/T*_*k*_.

**Figure 2:**
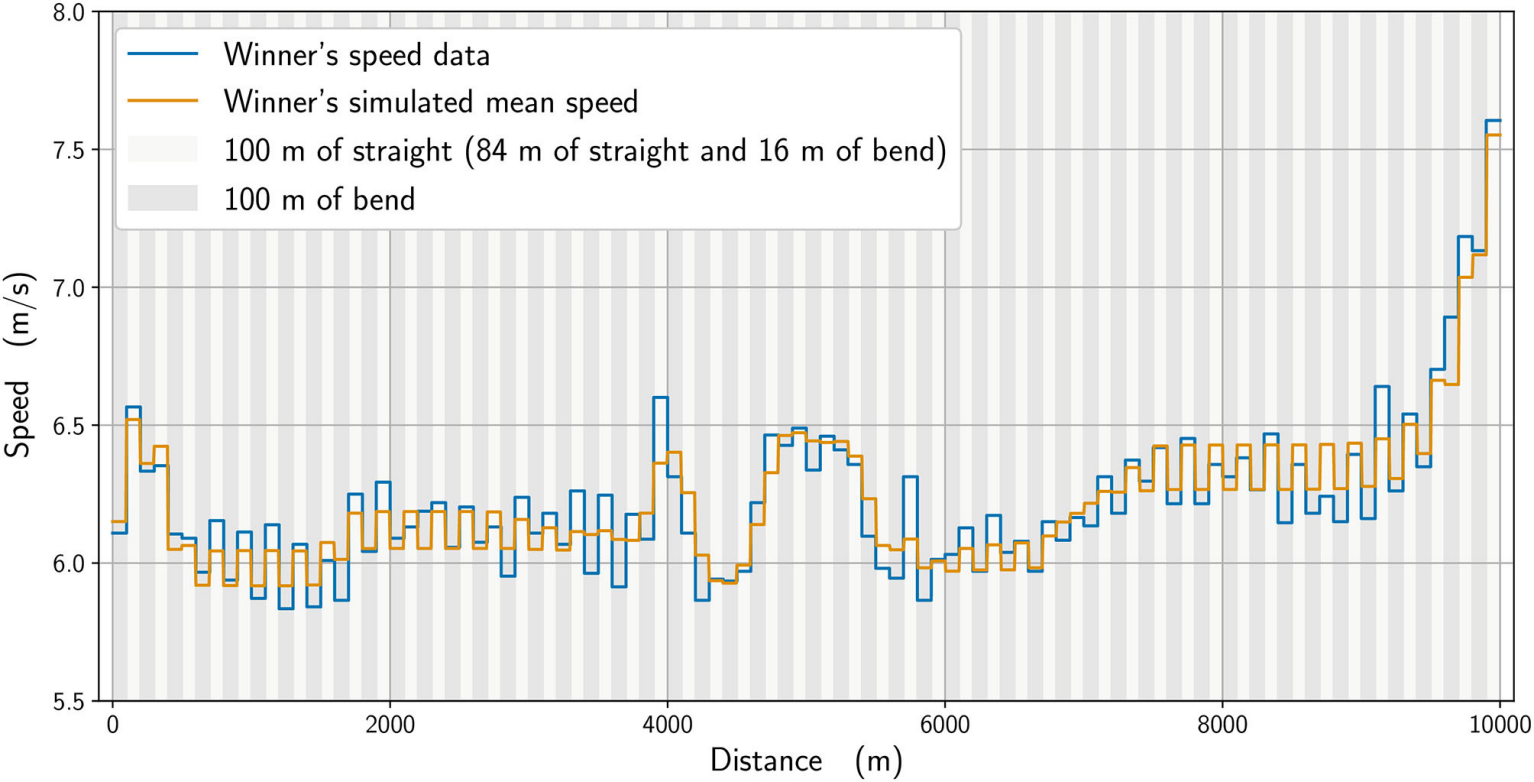
The winner's mean speed data (blue line) with final time *t*_data_ = 26:49.51 and the simulated mean speed (orange line) with final time *t*_simu_ = 26:48.92.

**Figure 3:**
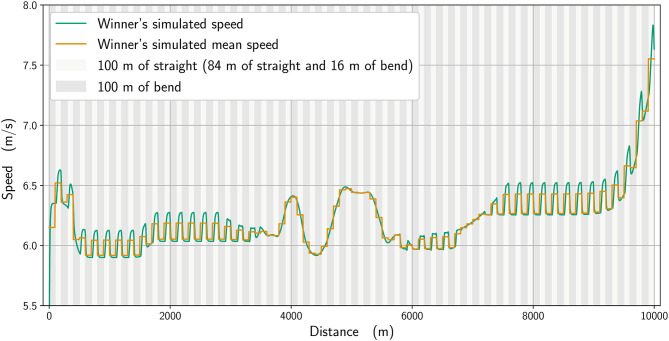
The simulated speed (green line) and its mean every 100 m (orange).

**Figure 4:**
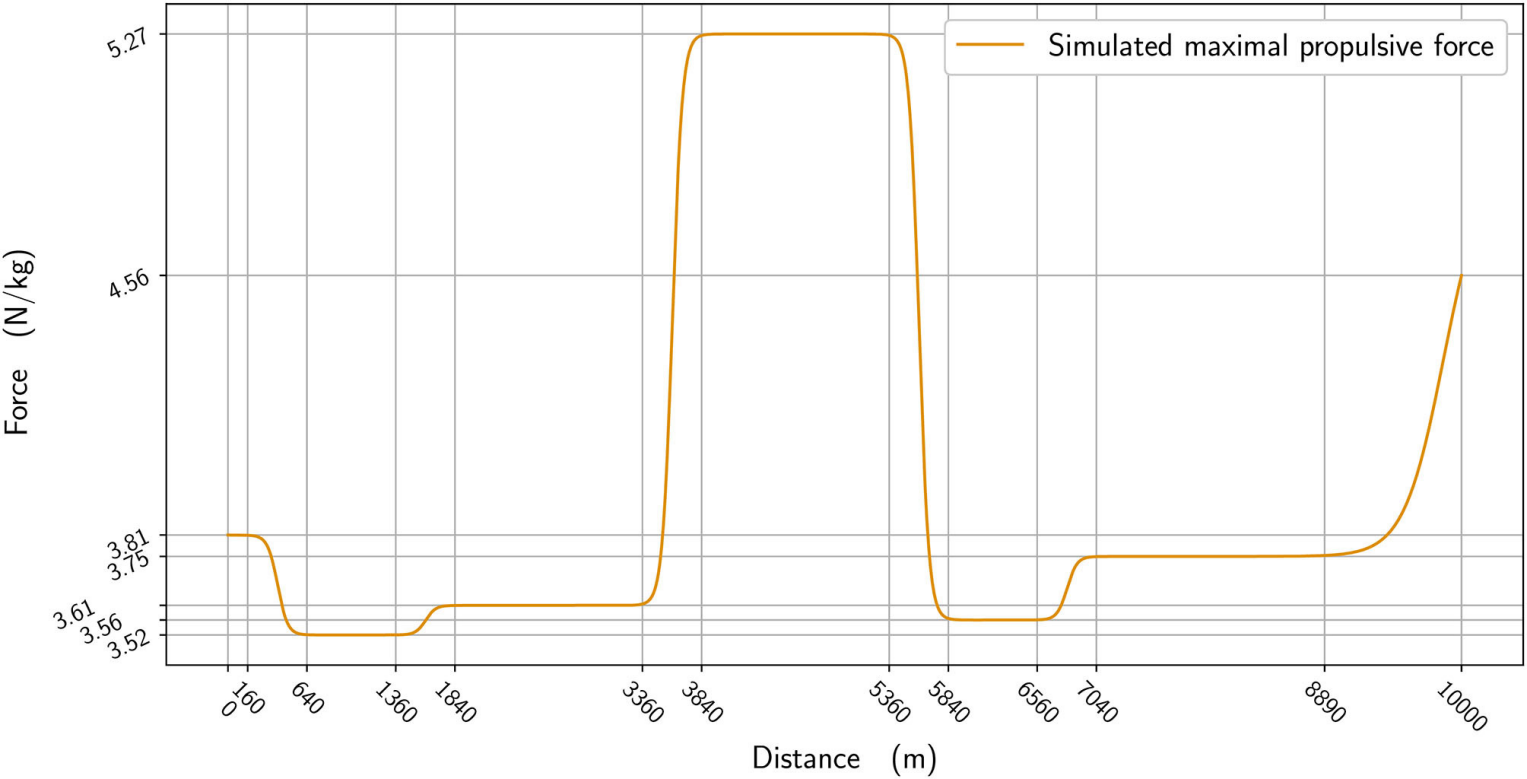
The maximal propulsive force per kg vs. distance.

**Figure 5:**
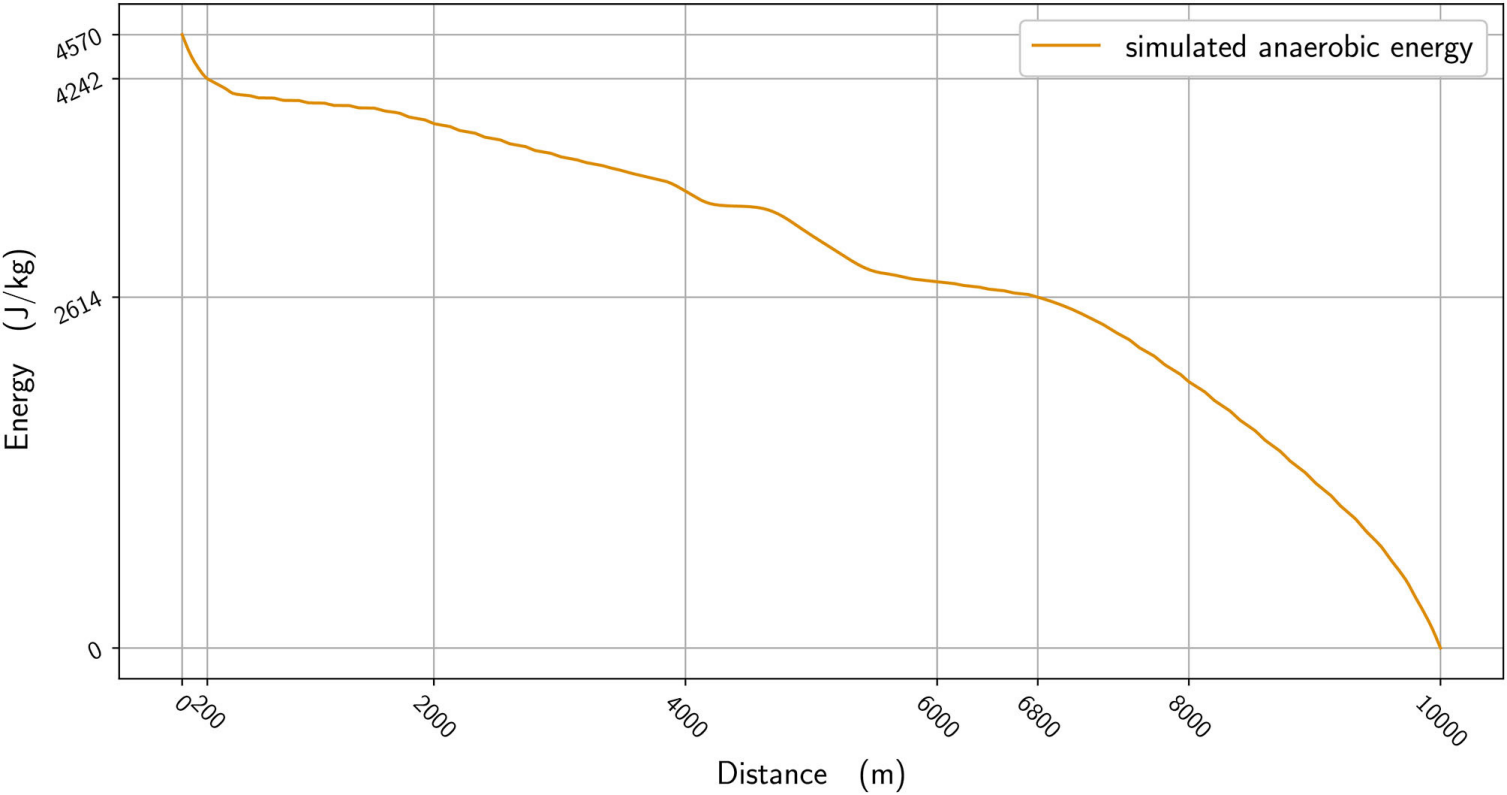
The anaerobic energy per kg vs. distance.

**Figure 6:**
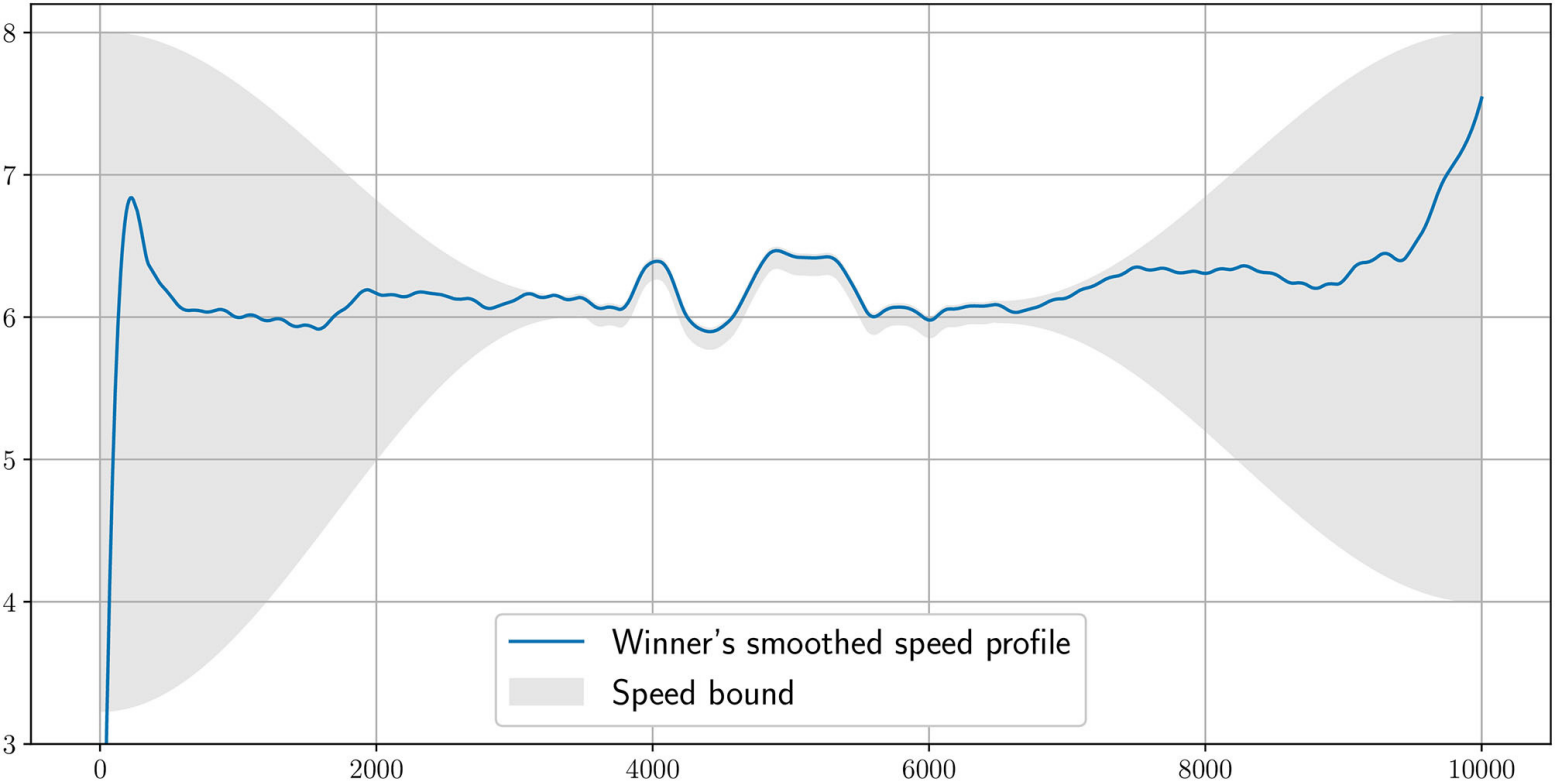
The speed bounds imposed in the simulation around the smoothed winner's speed profile. The speed has to be in the gray area. When the bounds are tight, this is to impose a strategic speed.

In Figure 2, we plot the winner's speed data (in blue) alongside the mean speed found from the simulation (orange), whereas in Figure 3, we add the instantaneous speed (green) to the simulated mean speed. On the instantaneous speed figure, we can observe that the instantaneous variations are much bigger on the bends (the gray vertical bands) than what we can surmise from the mean values. We also plot the simulated maximal propulsive force (Figure 4) to see how effort is organized during the race. Figure 5 provides the evolution of the anaerobic energy; we see that the consumption of anaerobic energy is more pronounced at the very beginning of the race and from 6,800 m for the final acceleration, ending with effective exhaustion of anaerobic reserve. The speed bounds are illustrated in Figure 6 where the strategic part can be seen clearly by the tightness of the bounds between ~4,000 and 6,000 m.

### Effect of the Parameters

**Figure 7:**
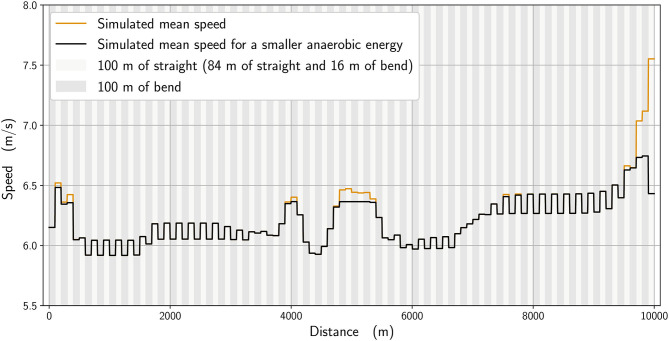
Simulated mean speed for the winner *t*_simu_ = 26:48.92 (orange line), and with 5% less energy (black line) *t*_less_ = 26:54.25, Δt = 5.33 s.

**Figure 8:**
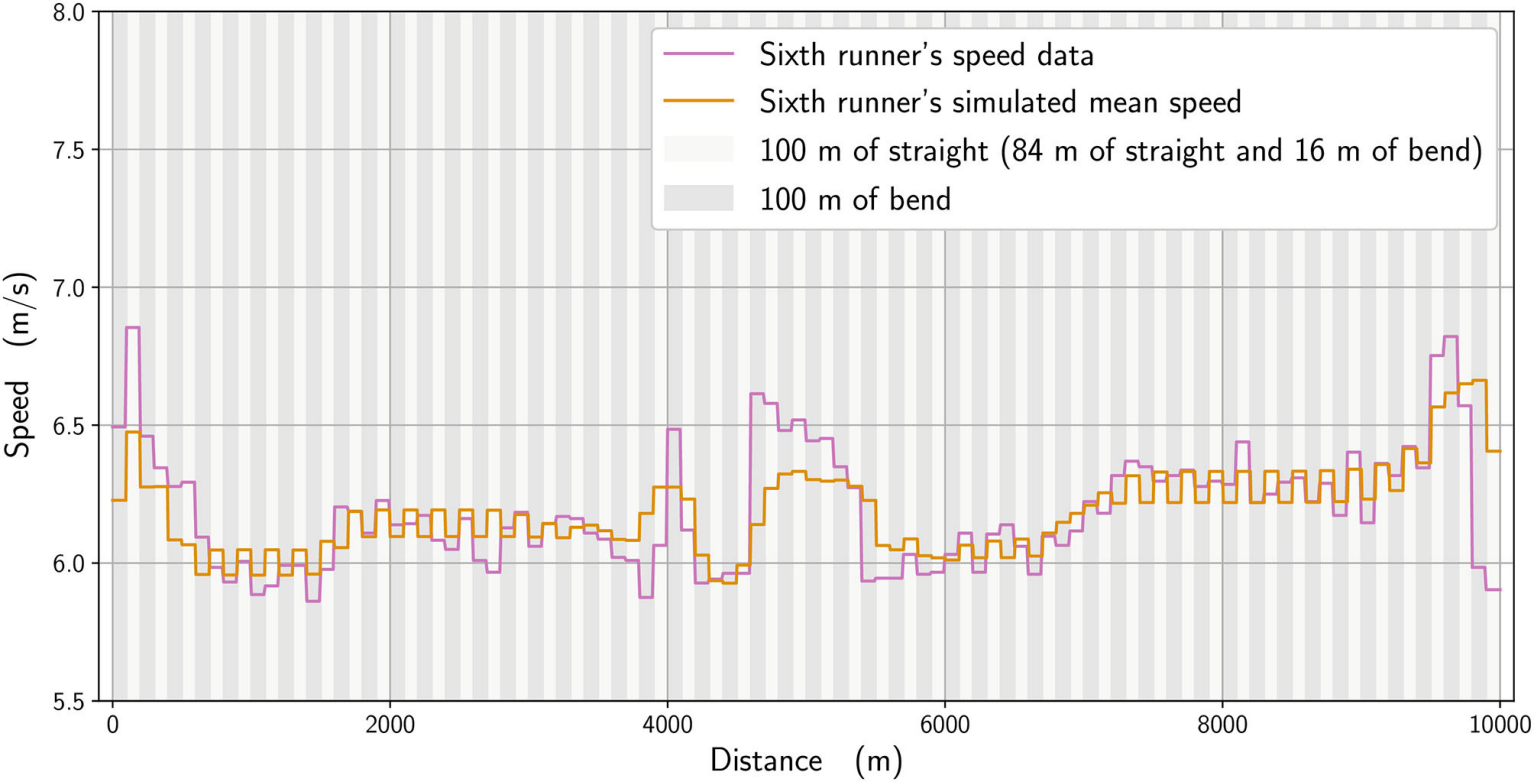
Simulated mean speed for a runner with a higher running economy (orange line) compared with the mean speed for the 6th-place runner (pink line). *t*_R6_= 26:57.92, *t*_simu_ = 26:57.41.

**Figure 9:**
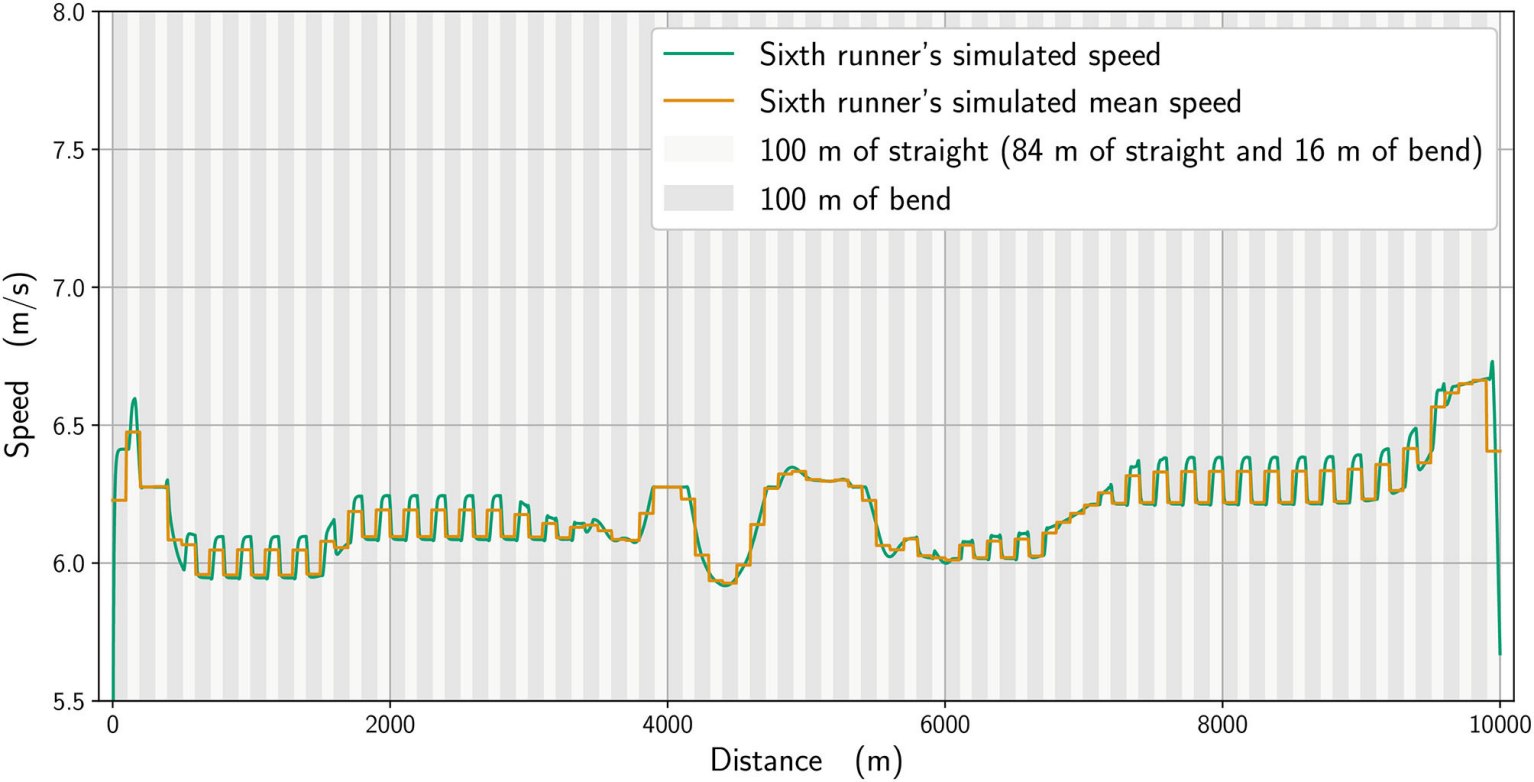
Simulated mean speed (orange line) and instantaneous speed for a runner with a higher running economy (green line).

We analyze numerically the effect of the physiological parameters. In Figure 7, we compare the winner's mean speed (in orange) with a simulation where the anaerobic energy is decreased by 5% (black), with the most noticeable effects occurring between about 4,800 and 5,400 m and from 9,600 m until the race finish. We subsequently select in our simulations an imaginary runner with a higher running economy (i.e., higher cost of running) (Lucia et al., 2008) and adjust his parameters to fit the 6th-place runner in the race. In Figure 8, we plot the mean speed data for the 6th-place runner (in pink), together with the simulated mean speed (orange), whereas in Figure 9, we plot the instantaneous speed (green). We see that the effect of these parameters is to strongly influence the strategic part of the race in the middle section and the final acceleration (or deceleration) at the end of the race.

## Discussion

Using the model, we can evaluate what factors influence race strategy, such as the ability to speed up during the endspurt. The simulation reproduces the main parts of the winner's race well (Figure 3): a strong acceleration at the beginning, followed by a cruising speed that is a little slower, a strategic acceleration between 4,000 and 6,000 m, then a slowing down to a slower speed before an acceleration starting at 7,000 m and a very quick last lap. The occurrence of a mid-race surge in speed is not unusual and was inferred to be a tactic used by the winner of the men's 10,000 m Olympic final in 2008 to increase the homeostatic disturbance in his opponents and effectively force them to choose a slower pace or risk dropping out (de Koning et al., 2011).

The 10,000 m race in this study was one that featured frequently observed aspects of racing, in that athletes did not implement an even pace strategy that would potentially result in better times, but instead adopted typical championship tactics such as varied pace and a fast endspurt. The closeness of the athletes with one lap remaining and, indeed, the narrow winning margin (0.43 s) meant that the winner needed to draw upon all remaining anaerobic reserves during the sprint finish. We see that the anaerobic energy reserve is used more in the sections of the race where a strong acceleration and propulsive force is required: at the very beginning; at around 4,000 m to speed up; and at the end (starting from 6,500 m) (Figure 5). Reasoning that the athletes finishing in the top positions in this race would have similar aerobic capacities, difference in anaerobic physiology can ultimately decide the final race positions. For the part of our analysis where we reduce the simulated winner's anaerobic energy by 5%, we see that the new optimal race has a start that is slightly slower, with an acceleration in the middle of the race that is smaller and, most important of all, a lack of ability to speed up at the end (Figure 7). If ever this hypothetical runner tried to speed up as much as the simulated winner at the beginning, then his pace would slow even more at the end. Coaches should therefore note the importance of developing sufficient anaerobic physiology in training, which is generally already understood but, more importantly, that athletes who are likely to be less well anaerobically trained should avoid the surges of increased speed that occur in the earlier stages of the race. These short surges are often deliberately used by athletes to challenge the physiological responses of their rivals (Thompson, 2007) and the model shows mathematically why this approach can work. By maintaining a more even pace throughout the race, any anaerobic energy that would be used up in these surges can be retained until the endspurt. In addition, an athlete who runs with a low “cruising” speed depletes their anaerobic energy less and enhances their capacity for a fast endspurt; this tactic can be seen in those distance runners with a fast sprint finish who try to deliberately slow the pace from the front, and is another strategy for coaches to consider training for.

There is an effect of bend running on speed, in that the modeled data (calculated to closely replicate the winner and 6th-place athletes' pacing profiles) indicate that the straights are run faster than the bends. This shows that an even pace is never truly achieved in 10,000 m racing, even during the more steady state phases of the race, e.g., between 1,000 and 3,000 m. Athletes do not immediately slow from a straight to a bend, not least because the 100-m straight sections themselves include 16 m of bend, and so the simulated speeds (Figures 3, 9) reflect the gradual decrease in pace on each bend and increase on the straights. To some extent, slower running speeds on the bends are not unexpected given the decrease in horizontal propulsion, and support previous assumptions by Taboga and Kram (2019) from their models of bend running. In any case, coaches should note that leg asymmetry is not uncommon during bend running, with the outer, right leg being more effective at force production (Judson et al., 2019). This means that a certain amount of preparatory training is required before competing in a 10,000 m track race (which comprises 5,800 m of bend running in total) to ensure both legs are effective in producing horizontal impulse and to avoid asymmetry-related injuries (Hamill et al., 1987). However, in the strategic parts between 4,000 and 6,000 m and near the end, when there is noticeable acceleration, the effect of the bend is less pronounced. We notice that for the 6th-place runner (Figure 9), the speed variation on the bends is not as great as for the winner: because he has a higher running economy, he cannot vary his speed as much in the straights after the bends. Moreover, he is unable to speed up at the end of the race as this inferior running economy means that maintaining a pace close to the winner relies more on anaerobic sources, which are then no longer available during the endspurt. In addition, a higher running economy reduces the ability to vary his speed once on the straight and restricts his tactical options more so than the athlete with lower running economy. Given that running economy could be the critical factor determining race performance (Lucia et al., 2008), it is undoubtedly a key factor for coaches to emphasize in training regimens (Midgley et al., 2007). It is reasonable to expect that psychological factors, such as demotivation caused by being out of the medal positions on the last lap, could have had an effect on the pacing profile of lower finishing runners. One strength of our model is the potential to include various psychological factors, either related to interactions between runners (Aftalion and Martinon, 2019), or motivation (or lack of motivation) using the cost-benefit model of Le Bouc et al. (2016). However, the difficulty of modeling these for a 10,000 m race is that the computations are very time-consuming and so for the moment we have not included these aspects. Nevertheless, for the 6th-place runner, we do not believe that reduced motivation or a decision to slow down explains the decrease in speed on the last lap because those finishing in the top eight have the incentive of prize money, and also because his speed decrease perfectly fits an energy reduction.

The advantage of using a modeling approach is that it allows us to manipulate the variables involved to assess the effect of changes in those variables, and thus the main strength of this study is how it accounts for the effect of each parameter individually and can be predictive. For example, by reducing anaerobic energy availability in our model we could see the reduced capacity for a fast endspurt or the need to avoid earlier intermittent surges of faster running. Being able to describe instantaneous speed for the whole race means we can account for more realistic changes in pace that using mean splits (over each 100 m) does not allow. One simple example is how the model allows us to show the rapid increase in speed from 0 m/s at the very start of the race, but more usefully means it is possible to see more clearly the effect of running the bends. In this study, we were interested in modeling championship performances, where tactics play a key role and where winning a medal is more important than the time achieved *per se*. Whereas, only a very few athletes can ever hope to set a world record (and planned attempts tend to require considerable race management on behalf of the organizers), championship running offers more athletes the chance to win a medal or achieve a high finishing position. A weakness of the modeling approach taken are that the computations for each race and runner are considerably time-consuming, and large-scale analyses of athletes and any possible interactions between them are costly. The results of this study show that coaches of ambitious athletes should consider training programs that emphasize both the ability to maintain a consistent pace for most of the race (e.g., through tempo runs) and the capability of increasing pace with no undue stress during tactical increases in speed and the endspurt (e.g., through short interval training) (Casado et al., 2020b). Further developments in athlete pace measurements that could provide higher resolution speed data (more frequently than each 100 m) will lead to better understanding, modeling and predictions that can assist coaches with race preparation. The implications for future research are therefore that how this knowledge allows an athlete to adapt a strategy against a runner whose weakness or parameters are known could be tested; indeed, the practical applications of this study include the ability for coaches to adapt race strategy based on knowledge of how energy reserves are used, as well as highlighting the importance of becoming proficient at bend running.

## Conclusions

Based on 100-m time splits for a 10,000 m race, we perform simulations to understand the role of the physiology, efforts and tactics, and what is essential to win a race. We have seen that a runner with a low running economy can speed up in the middle of the race and maintain a strong pace that will not impact his ability to speed up considerably at the end. On the other hand, a runner with a higher running economy or with a lower anaerobic reserve, or where higher running economy is compensated for with anaerobic energy, can follow most of the race but loses the ability to speed up at the end. We also observe that runners with better running economy have the ability to vary more their speed between the bends and straights, leading to a better time per lap, and overall race performance. Although it is well-established that physiological factors like running economy and anaerobic energy are important in elite-standard endurance running, we show for the first time how athletes who are more limited in these aspects can reduce the impact by adopting race tactics such as even pace running until the final acceleration phase. In addition, there is a clear effect of the bends on running speeds, and athletes are advised to spend some time in preparation for 10,000 m races to familiarize themselves with bend running at race speeds.

## Data Availability Statement

The raw data supporting the conclusions of this article will be made available by the authors, without undue reservation.

## Author Contributions

AA, BH, and QM conceptualized and designed the study. AA and BH wrote the manuscript. AA and QM conducted the data analyses and created the figures. All authors read and approved the final manuscript.

## Conflict of Interest

The authors declare that the research was conducted in the absence of any commercial or financial relationships that could be construed as a potential conflict of interest.

## References

[B1] Aftalion A. (2017). How to run 100 meters. SIAM J. Appl. Math. 77, 1320–1334. 10.1137/16M1081919

[B2] Aftalion A. Bonnans J. F. (2014). Optimization of running strategies based on anaerobic energy and variations of velocity. SIAM J. Appl. Math. 74, 1615–1636. 10.1137/130932697

[B3] Aftalion A. Martinon P. (2019). Optimizing running a race on a curved track. PLoS ONE 14:e0221572. 10.1371/journal.pone.0221572 31487301 PMC6728027

[B4] Aftalion A. Trélat E. (2020). How to build a new athletic track to break records. R. Soc. Open Sci. 7:200007 10.1098/rsos.200007 32269819 PMC7137942

[B5] Billat V. Lepretre P. M. Heugas A. M. Laurence M. H. Salim D. Koralsztein J. P. (2003). Training and bioenergetic characteristics in elite male and female Kenyan runners. Med. Sci. Sports Exerc. 35, 297–304. 10.1249/01.MSS.0000053556.59992.A9 12569219

[B6] Bonnans J. F. Giorgi D. Grelard V. Maindrault S. Martinon P. (2014). BOCOP—A Toolbox for Optimal Control Problems. Available online at: http://bocop.org (accessed November 24, 2020).

[B7] Burnley M. Jones A. M. (2010). 'Traditional' perspectives can explain the sprint finish. In: Comments on Point:Counterpoint: Afferent feedback from fatigued locomotor muscles is/is not an important determinant of endurance exercise performance. J Appl. Physiol. 108, 458–468. 10.1152/japplphysiol.01388.2009

[B8] Casado A. Hanley B. Jiménez-Reyes P. Renfree A. (2020a). Pacing profiles and tactical behaviors of elite runners. J. Sport Health Sci. 10.1016/j.jshs.2020.06.011. [Epub ahead of print]. PMC8500812 32599344

[B9] Casado A. Hanley B. Ruiz-Pérez L. M. (2020b). Deliberate practice in training differentiates the best Kenyan and Spanish long-distance runners. Eur. J. Sport Sci. 20, 887–895. 10.1080/17461391.2019.1694077 31724902

[B10] Churchill S. M. Trewartha G. Salo A. I. (2019). Bend sprinting performance: new insights into the effect of running lane. Sports Biomech. 18, 437–447. 10.1080/14763141.2018.1427279 29562837

[B11] de Koning J. J. Foster C. Bakkum A. Kloppenburg S. Thiel C. Joseph T.. (2011). Regulation of pacing strategy during athletic competition. PLoS ONE 6:e15863. 10.1371/journal.pone.0015863 21283744 PMC3024328

[B12] Filipas L. La Torre A. Hanley B. (2018). Pacing profiles of Olympic and IAAF World Championship long-distance runners. J. Strength Cond. Res. 10.1519/JSC.0000000000002873. [Epub ahead of print]. 30289868

[B13] Foster C. de Koning J. J. Hettinga F. Lampen J. Dodge C. Bobbert M.. (2004). Effect of competitive distance on energy expenditure during simulated competition. Int. J. Sports Med. 25, 198–204. 10.1055/s-2003-45260 15088244

[B14] Hamill J. Murphy M. Sussman D. (1987). The effects of track turns on lower extremity function. Int. J. Sport. Biomech. 3, 276–286. 10.1123/ijsb.3.3.276

[B15] Hanley B. Bissas A. Merlino S. (2018). “Biomechanical report for the IAAF World Championships London 2017: 10,000 m men's,” in 2017 IAAF World Championships Biomechanics Research Project, July 2018 (London). Monte Carlo: IAAF. Available online at: https://www.worldathletics.org/about-iaaf/documents/research-centre (accessed November 24, 2020).

[B16] Hettinga F. J. Edwards A. M. Hanley B. (2019). The science behind competition and winning in athletics: using world-level competition data to explore pacing and tactics. Front. Sports Act. Living 1:11. 10.3389/fspor.2019.00011 33344935 PMC7739697

[B17] Jones A. M. Kirby B. S. Clark I. E. Rice H. M. Fulkerson E. Wylie L. J.. (2020). Physiological demands of running at 2-hour marathon race pace. J Appl. Physiol. 10.1152/japplphysiol.00647.2020. [Epub ahead of print]. 33151776

[B18] Jones A. M. Whipp B. J. (2002). Bioenergetic constraints on tactical decision making in middle distance running. Br. J. Sports Med. 36, 102–104. 10.1136/bjsm.36.2.102 11916890 PMC1724468

[B19] Joyner M. J. (1991). Modeling: optimal marathon performance on the basis of physiological factors. J. Appl. Physiol. 70, 683–687. 10.1152/jappl.1991.70.2.683 2022559

[B20] Judson L. J. Churchill S. M. Barnes A. Stone J. A. Brookes I. G. Wheat J. (2019). Horizontal force production and multi-segment foot kinematics during the acceleration phase of bend sprinting. Scand. J. Med. Sci. Sports 29, 1563–1571. 10.1111/sms.13486 31131939

[B21] Le Bouc R. Rigoux L. Schmidt L. Degos B. Welter M. L. Vidailhet M.. (2016). Computational dissection of dopamine motor and motivational functions in humans. J. Neurosci. 36, 6623–6633. 10.1523/JNEUROSCI.3078-15.2016 27335396 PMC6601748

[B22] Lucia A. Oliván J. Bravo J. Gonzalez-Freire M. Foster C. (2008). The key to top-level endurance running performance: a unique example. Br. J. Sports Med. 44, 172–174. 10.1136/bjsm.2007.040725 18048445

[B23] Midgley A. W. McNaughton L. R. Jones A. M. (2007). Training to enhance the physiological determinants of long-distance running performance. Sports Med. 37, 857–880. 10.2165/00007256-200737100-00003 17887811

[B24] Ohnuma H. Tachi M. Kumano A. Hirano Y. (2018). How to maintain maximal straight path running speed on a curved path in sprint events. J. Hum. Kinet. 62, 23–31. 10.1515/hukin-2017-0175 29922374 PMC6006540

[B25] Padilla S. Mujika I. Angulo F. Goiriena J. J. (2000). Scientific approach to the 1-h cycling world record: a case study. J. Appl. Physiol. 89, 1522–1527. 10.1152/jappl.2000.89.4.1522 11007591

[B26] Péronnet F. Thibault G. (1989). Mathematical analysis of running performance and world running records. J. Appl. Physiol. 67, 453–465. 10.1152/jappl.1989.67.1.453 2759974

[B27] Pritchard W. G. (1993). Mathematical models of running. SIAM Rev. 35, 359–379. 10.1137/1035088

[B28] Quinn M. D. (2003). The effects of wind and altitude in the 200-m sprint. J. Appl. Biomech. 19, 49–59. 10.1123/jab.19.1.4915801501

[B29] Quinn M. D. (2009). The effect of track geometry on 200- and 400-m sprint running performance. J. Sports Sci. 27, 19–25. 10.1080/02640410802392707 18979339

[B30] Renfree A. Casado A. Pellejero G. Hanley B. (2020). More pace variation and pack formation in successful world-class 10,000-m runners than in less successful competitors. Int. J. Sports Physiol. Perform. 15, 1369–1376. 10.1123/ijspp.2019-0852 32957080

[B31] Saltin B. Larsen H. Terrados N. Bangsbo J. Bak T. Kim C. K.. (1995). Aerobic exercise capacity at sea level and at altitude in Kenyan boys, junior and senior runners compared with Scandinavian runners. Scand. J. Med. Sci. Sports 5, 209–221. 10.1111/j.1600-0838.1995.tb00037.x 7552766

[B32] Taboga P. Kram R. (2019). Modelling the effect of curves on distance running performance. PeerJ 7:e8222. 10.7717/peerj.8222 31879575 PMC6927354

[B33] Thiel C. Foster C. Banzer W. de Koning J. (2012). Pacing in Olympic track races: competitive tactics versus best performance strategy. J. Sports Sci. 30, 1107–1115. 10.1080/02640414.2012.701759 22738897

[B34] Thompson P. J. L. (2007). Perspectives on coaching pace skill in distance running: a commentary. Int. J. Sports Sci. Coach. 2, 219–221. 10.1260/174795407782233128

[B35] World Athletics (2019a). Track and Field Facilities Manual. Monte Carlo: World Athletics.

[B36] World Athletics (2019b). C2.1—Technical Rules. Monte Carlo: World Athletics.

[B37] World Athletics (2020a). Details of Cheptegei's 10,000m World Record Assault Revealed. Available online at: https://www.worldathletics.org/news/news/cheptegei-10000-wr-assault-valencia-details (accessed November 17, 2020).

[B38] World Athletics (2020b). Results−100 metres Men Heats. Available online at: https://www.worldathletics.org/competitions/world-athletics-championships/iaaf-world-championships-london-2017-7093740/results/men/100-metres/heats/result (accessed December 17, 2020).

